# Efficiency of high-carbohydrate fodder in the diets of Holstein cows

**DOI:** 10.14202/vetworld.2021.1303-1310

**Published:** 2021-05-25

**Authors:** Irina Mironova, Alexey Pleshkov, Azat Nigmatyanov, Elvira Yarmukhamedova, Sofia Islamova

**Affiliations:** 1 Federal State Budgetary Educational Establishment of Higher Education “Bashkir State Agrarian University”, Ufa, Russia; 2 Federal State Budgetary Educational Institution of Higher Education Ufa State Petroleum Technological University (USPTU), Ufa, Russia

**Keywords:** animal breeding, cows, diet, ration cuts

## Abstract

**Aim:**

This study aimed to analyze the efficiency of carbohydrate-enriched rations fed to pasture and stall-housed cows.

**Materials and Methods:**

Forty Holstein cows were divided into four groups of 10 animals each. The experiment lasted 305 days. All animals were kept under the same conditions, except for the amount of energy-carbohydrate rations fed. The control cows were on a standard diet. Experimental groups 1, 2, and 3 received a ration enriched with energy-carbohydrate components at 250, 500, and 700 g/head/day, respectively. Feed intake was measured on 2 consecutive days each month. During the balance trial, when young animals reached 13 months, feed intake was examined daily. Fodder, its residues, feces, and urine collected during the balance trial were used to determine nutrient digestibility and nitrogen exchange.

**Results:**

Nitrogen balance was positive in all experimental animals. Cows in Group 3 made better use of the digested nitrogen. A biochemical blood test showed higher total protein content in the serum of the experimental cows than in the control by 1.47-3.49% (p≤0.05-0.001) in winter and 0.24-0.98% (p≤0.01) in summer. In winter, the serum protein level increased due to changed feeding routines and shorter exercise times, but did not exceed physiological requirements. The alpha- and gammα-globulins levels increased to 0.2-0.5 g/L in winter and 0.3-0.6 g/L in summer, and 0.5-1.4 g/L in winter and 0.1-0.2 g/L in summer, respectively. Beta-globulins decreased. The supplement had a positive effect on milk output, which increased by 67.1-137.3 kg (1.93-3.95%; p≤0.05-0.001) in the first 100 lactation days, then by 198.2-458.8 kg (2.39-5.53%; p≤0.05-0.001) for the remaining days. All animals had a high milk yield coefficient, with the experimental groups having 0.64-2.64% more milk yield than the control. The lactation curves showed that the average daily milk yield of all experimental cows increased, then gradually decreased along the physiological trajectory from the 3^rd^ month until the end of lactation. Milk quality analysis in the experimental groups indicated an increase in the dry matter content by 0.17-0.27% (p≤0.001) in summer and 0.16-0.27% (p≤0.001) in winter; higher protein levels by 0.04-0.06% in summer and 0.03-0.07% (p≤0.05) in winter; increased fat by 0.09-0.14% (p≤0.05-0.001) in summer and 0.09-0.13% (p≤0.05-0.001) in winter; increased density by 0.47-0.61°A (p≤0.05) in summer and 0.17-0.27°A in winter; and increased energy by 1.70-2.63% (p≤0.001) in summer and 1.57-2.54% (p≤0.01-0.001) in winter.

**Conclusion:**

The energy-carbohydrate feed “Tanrem” can provide the required energy intake of Holstein cows. The maximum biological and economic effect wads attained at 500 g per animal.

## Introduction

Holstein cattle account for 67% of the total cattle population in the Russian Federation. These are large, highly productive animals capable of producing large quantities of milk. The intensive growth and development of young Holstein cattle make the breed particularly interesting for milk production. Although some improvements in the milk yield have been achieved, further rise can be attained by improving the breed’s typically low feed conservation ratios [1-4]. One of the valuable breed qualities of Holstein cattle is the intensive growth and development of juveniles. Improvements in breed-specific traits could increase the breed’s genetic potential for milk productivity and improve the breed’s suitability for industrial maintenance, since the husbandry requirements for Holstein cattle are very high. If these needs are not met (i.e., only the physiological needs are fulfilled), then the breed suffers from low feed conversion ratios and their genetic potential is not attained [5].

Previous research has shown that food additive enriched diets increase livestock productivity. Such diets must be formulated to meet the animal’s nutritional needs, while also considering the feed’s safety, cost, and preferably waste-free production [6,7]. The diet formulation requires a thorough selection of fodders to transform the necessary amount of nutrients and energy into the animal’s body [8-10]. During lactation, dairy cattle must consume enough energy and nutrients, since the body’s glucose requirements during this period are 3 times higher than average due to milk production [11,12]. Farmers often address this problem by increasing the proportion of concentrates, oilseed residues, cakes, and grain fodder; however, these feeds also increase the blood’s protein content, resulting in health problems, such as ketosis, acidosis, and reproductive disorders [13].

In recent years, scientists and practitioners have become interested in energy supplements as a means to prevent balance problems, weight reduction, metabolic abnormalities, and reproductive disorders [4,14,15]. A wide assortment of energy supplements in a variety of formulations is currently available for livestock. The most widely used additive is propylene glycol. With a daily dosage of 100-300 g per animal, it improves the digestibility of dry and organic matter, and increases milk yields. When given with glycerin and niacin, these improvements are increased further. The same effect can be achieved using protected fat (300 g/animal/day), hepatoprotectors in the form of protected B vita­mins [16-18].

When increasing milk output, it is important to assess the cow’s protein metabolism, since it is involved in all body functions. Digestive enzymes break down feed protein into polypeptides and amino acids, which are then absorbed into the blood. Nitrogen, a building block of organic matter, can be used to evaluate the feed intake amounts. Nitrogen balance can be positive, negative, or zero and can be used to estimate the digested protein weight, as well as the potential increase or decrease in the animal’s protein [19]. Vital body functions depend on biochemical processes that affect product synthesis. Changes in blood composition indicate metabolism intensity and the associated processes of growth, development, and productivity. Thus, biochemical blood tests were used to analyze feed efficiency and potential productivity [20].

Animals with similar heredity develop differently under the influence of varied environmental conditions [2,21]. This study aimed to analyze the efficiency of carbohydrate-enriched rations fed to pasture and stall-housed cows.

## Materials and Methods

### Ethical approval

The animals were treated in accordance with the instructions and guidelines of Russian Regulations, 1987 (Order No. 755 on 12.08.1977 the USSR Ministry of Health) and “The Guide for Care and Use of Laboratory Animals” (National Academy Press Washington, D.C. 1996)” The number of testing samples was minimal.

### Study period and location

The study was conducted during 2019 and 2020 in Agroalliance LLC, located in the Chishmy district of the Bashkortostan Republic.

### Research target

The experiment was carried out in the South Ural climate and lasted for 305 lactation days. A total of 40 Holstein cows were used in the research. The cows were selected by analogy from the cattle raised on the farm. The animals were kept at pasture for 6 months from the start of the experiment. As the weather worsened, the cattle were kept indoors. All animals were kept in the same conditions and the cows were allocated into four groups: A control group and three experimental groups. The control group received a standard diet, while the experimental animals in Groups 1, 2, and 3 were fed diets enriched with carbohydrates at a daily dose of 250, 500, and 700 g/animal/day, respectively. The feed supplement differed from other additives in that it had easy hydrolysable carbohydrates and zero non-protein, nitrogenous compounds.

The cow diets were formulated differently for indoor versus pasture use and were analyzed independent of the four groups. At pasture, the cow diets consisted of 80.4-86.9% succulent fodder and 12.2-13.1% concentrates. During indoor maintenance, the cows were fed 24.3-25.9% succulent feed, 34.4-36.7% concentrates, and 35.0-37.4% roughages. The succulent fodder share decreased with addition of the tested additive. The pasture ration for all groups consisted of grass, legumes, barley, oats, peas, table salt, and monosodium phosphate. During the stall feeding period, the grass was replaced with alfalfa haylage, barley straw, corn silage, molasses, and a pre-mixed P60-1 additive. Juvenile animals in the groups were fed diets enriched with the “Tanrem” additive at a dose of 0.25; 0.50; and 0.75 g for Groups 1-3, respectively. The rations were formulated based on milk productivity level, the physiological state of the cows, and feed quality and were occasionally adjusted. The diet was balanced in a program designed to calculate nutritional needs to plan the appropriate preparation and consumption of the feed during different cattle maintenance periods.

### Preparation

Treatments were based on a new, high-carbohydrate energy additive that was introduced in different dosages in the feed mixture and fed in twice daily: In the morning and at 2-3 o’clock in the afternoon. The distinctive feature of the feed additive Tandem (Kapital-Prok; Moscow region, Balashikha) is its low protein content and high level of extended-release energy, which is essential for cows after calving to increase milk production and weight gain. The supplement developers recommend using this additive instead of molasses, protected fats, and propylene glycol. The additive contains at least 50% easily digestible carbohydrates, including up to 27% sucrose, 8.0% vegetable protein, and 12% fat. The studied feed is a fine powder of uniform consistency and is light yellow to dark brown in color. The supplement consists of fibers aimed at activating rumen motility. The developers made it more palatable with a chocolate taste and flavor for better intake and absorption. The ingredients include natural prebiotics and energy additives, which activate metabolic processes.

### Diagnostic methods

The biochemical blood parameters were measured using conventional methods. The blood samples were taken from the jugular vein of healthy cows in the morning before feeding and watering. Specifically, biosubstrates were collected into vacuum tubes with a coagulation activator (thrombin). Biochemical analysis was performed with an automatic analyzer CS-T240 (Dirui Industrial Co., Ltd., China) using the commercial veterinary kits, DiAvTest (Russia) and Randox Laboratories Limited (UK).

The digestion trial involved preparatory (3 days), preliminary (15 days), and accounting (10 days) periods. Three representative animals from each group were selected. During the preliminary period, the remnants from previous feed were removed from the gastrointestinal tract of the experimental animals, then were filled with the tested feed additive. During the accounting period, the amount of feed consumed (including residues) and the amount of feces excreted were recorded. Then, the fodder and feces were analyzed for moisture, dry matter, protein, fat, fiber, nitrogen-free extractive substances, and minerals. Digested substances were calculated as the difference between their average daily intake and their average excretion in feces.

The difference between the amount of nitrogen ingested and the amount of nitrogen excreted from the body is called the nitrogen balance. Nitrogen balance measurement involves the analyses of the digestibility of nitrogenous substances in the diet and nitrogen exchange. In other words, a common digestion trial is supplemented by urine collection and nitrogen content calculation. The total nitrogen excretion per day is measured based on the quantitative and chemical analysis of the excreted feces and urine. When conducting experiments with lactating cows, the nitrogen content in the daily milk yield is also considered. Nitrogen balance can be used to determine the amount of protein accumulated or broken down (in negative nitrogen balance). Nitrogen balance was calculated based on the balance experiment data. During this period, the weight of the specified feed and their residues was measured, as was urine, feces, and milk output. All samples for laboratory testing were preserved. Feces from animals were collected in enameled tanks, then filled with 10% hydrochloric acid at the rate of 50 ml per 1 kg of feces and add 2 ml of chloroform. The storage box with feces was kept in the cold. Every day, the feces were weighed, mixed well, taken 1-2 % by weight of feces from different places and placed in jars with ground stoppers. Fecal samples were preserved by adding 100 ml of 10% hydrochloric acid solution and 2 ml of chloroform per 1 kg of feces. The samples were stored in a cool place before analysis. Urine, as excreted by animals, was collected in a prepared bottle with pre-poured 10-15 cm^3^ of a 10% hydrochloric acid solution and 2-3 g of thymol. Ten percentage of average urine samples were taken from the daily urine excretion and placed in bottles with ground stoppers. The samples were additionally preserved with a 10% solution of hydrochloric acid, so that the total amount of added acid was 5 % of the sample weight. Then 2-3 g of thymol was added once or twice during the experiment. The samples were stored at a temperature of 3-5°C until the end of the analysis. In lactating animals, milk was recorded and average samples were taken for analysis at each milking. The milk samples were approximately 1-2 % of the milk yield. The minimum daily sample size was 100 ml. The milk was preserved with formalin (8 drops per 1 liter of milk).The nitrogen balance was determined by the formula:

Feed nitrogen=Feces nitrogen+Urine nitrogen+Nitrogen retention.

Milk productivity was evaluated for 305 days of lactation according to monthly control milking. Test milking is used to evaluate daily milk productivity, fat, and protein percentages. Lactation curves for the control and experimental groups were constructed. Milk production per month was calculated by the daily milk yield multiplied by 30. The milk yield coefficient was calculated based on the milk output for 305 days, the cows’ live weight, the stability index, and the lactation efficiency [22]. The lactation efficiency was calculated by the formula: LE = (TMY/[HDY×n])·100, where LE is the lactation efficiency; TMY is the total milk yield for lactation; HDY is the highest daily yield; and n is the lactation length. The lactation stability index was determined by: LSI = (MY2/MY1)*100, where MY1 is the milk yield for the first 90 days of lactation; and MY2 is the milk yield for the second 90 days of lactation.

### Statistical analysis

Standard animal examination methods for the biometric processing of test samples were used supported with Microsoft Excel-2010 software (Microsoft, USA). The difference in mean indicators was considered reliable per the Student’s criterion (at p < 0.05; p < 0.01; and p < 0.001). We focused on the feeding factor to determine its effect on productivity, thus, the normal distribution was not checked before the *t*-test.

## Results

**Figure-1:**
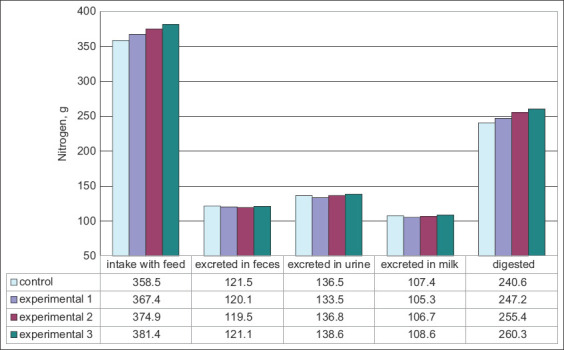
Nitrogen balance in cows, n=3.

The average daily nitrogen balance was positive in all experimental animals (Figure-1). The nitrogen intake in the experimental groups was increased by 8.9-23.0 g (2.5-6.4%; p≤0.05-0.01). The highest level of excreted nitrogen in the feces was in the control group, with values 0.4-2.0 g (0.3-1.7%) more than in the experimental groups. Most of all, total nitrogen was released with feces, urine, and milk in the control group, which exceeded the experimental animals by 4.6-13.8 (1.3-3.9%) with an unreliable difference. The nitrogen utilization in the control group was 30.2% of their total nitrogen intake, which is 0.8-1.7% lower than in the experimental groups. In addition, the nitrogen utilization from the digested was 38.1% in the control animals, which is lower than in the experimental cows by 0.5-1.7%. The control animals used more nitrogen for milk production (29.1%) than they consumed, which was 0.4-0.6% higher than the experimental groups. The control group used 43.3% of their digested nitrogen, which was 0.7-1.6% more than the experimental groups.

The high-carbohydrate energy supplement had a positive effect on the biochemical homeostasis of the experimental animals (Table-1). Thus, relative to the control animals, the experimental Holstein cows had an increased total protein content by 1.1-2.6 g/L (1.47-3.49%; p≤0.05-0.001) in winter and 0.2-0.8 g/L (0.240.98%; p≤0.01) in summer. The albumin and globulin content in all seasons had similar results to the total protein levels. In winter, the concentration of serum albumin in the control cows was 36.0 g/L, which was higher by 1.7-3.6% and 0.49-1.23% than the experimental groups. The reliability of these results was confirmed by the second and third experimental groups in all seasons of the year. The blood serum globulin fractions consisted of alpha-, beta-, and gamma-globulins. The alpha- and gamma-globulin levels in the blood of the experimental groups tended to be higher. Alpha-globulins were more by 0.2-0.5 g/l in winter and by 0.3-0.6 g/l in summer, while gamma-globulins were more by 0.5-1.4 g/l and 0.1-0.2 g/l, respectively. The beta-globulin levels in the control group compared to the experimental ones was higher both in winter and summer

**Table-1:** Protein composition of blood serum, g/L (X±Sx).

Indicator	Season	Group			

		Control	Experimental 1	Experimental 2	Experimental 3
Total protein	Winter	74.6±0.24	75.7±0.30*	77.2±0.23***	77.1±0.57**
	Summer	81.9±0.16	82.1±0.12	82.7±0.19**	82.7±0.18**
Albumins	Winter	36.0±0.30	36.6±0.34	37.3±0.40*	37.3±0.40*
	Summer	40.5±0.06	40.7±0.08	41.0±0.14*	41.0±0.16*
Globulins	Winter	38.6±0.49	39.1±0.51	39.9±0.17*	39.9±0.94
	Summer	41.4±0.11	41.4±0.17	41.7±0.12	41.7±0.28
Alpha	Winter	10.5±0.26	10.7±0.31	11.0±0.05	10.9±0.08
	Summer	10.0±0.27	10.3±0.33	10.6±0.30	10.5±0.40
Beta	Winter	11.5±0.29	11.2±0.21	10.8±0.18	11.0±0.26
	Summer	11.1±0.07	10.8±0.14*	10.7±0.28	10.8±0.34
Gamma	Winter	16.7±0.84	17.2±0.76	18.1±0.28	17.9±0.70
	Summer	20.3±0.43	20.4±0.61	20.4±0.46	20.5±0.84
Albumin-globulin coefficient	Winter	0.93±0.02	0.93±0.02	0.94±0.01	0.94±0.03
	Summer	0.98±0.01	0.98±0.01	0.98±0.01	0.98±0.01

The new energy supplement increased cattle milk productivity in the first 100 days of lactation (Table-2). The experimental cows exceeded the control cows in terms of milk output. For the first 100 days of lactation, the milk yields in the experimental cows were increased by 67.1 kg (1.93%; p≤0.05) in Group 1; by 137.3 kg (3.95%; p≤0.001) in Group 2; and by 123.8 kg (3.56%; p≤0.001) in Group 3. For the entire 305 days period, milk yield was higher by 198.2 kg (2.39%; p≤0.05) for Group 1; 458.8 kg (5.53%; p≤0.001) for Group 2; and 408.9 kg (4.93%; p≤0.001) for Group 3.

**Table-2:** Milk production.

Indicator	Group			

	Control	Experimental 1	Experimental 2	Experimental 3
Milk yield for 305 days in milk, kg	8292.4±69.93	8490.6±67.73*	8751.2±42.24***	8701.3±46.06***
Milk yield for 100 days in milk, kg	3473.0±18.75	3540.1±22.15*	3610.3±17.38***	3596.8±18.79***
Average daily milk yield, kg	27.2±0.23	27.8±0.22*	28.7±0.14***	28.5±0.15***
Milk yield coefficient, kg	1572.0±12.29	1582.0±14.30	1613.5±10.71*	1613.5±10.88*

One of the most significant indicators for the assessment of milk productivity is the milk yield coefficient. Its value indicates the mass of milk produced per 100 kg live weight of the animal. This coefficient can indirectly demonstrate the metabolic pathways in the animal body, including the productivity traits of the livestock. A high milk yield coefficient was found for all experimental cows, which indicates their pronounced dairy type. Simultaneously, a comparative analysis between the groups showed that the milk yield coefficient in the experimental animals was 10.0-41.5 kg (0.64-2.64%) higher than in the control cows.

**Figure-2:**
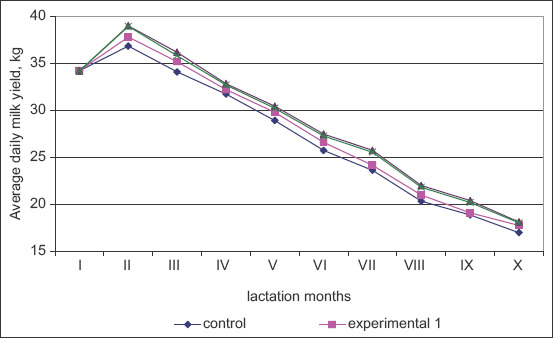
Lactation curves by lactation months.

**Figure-3:**
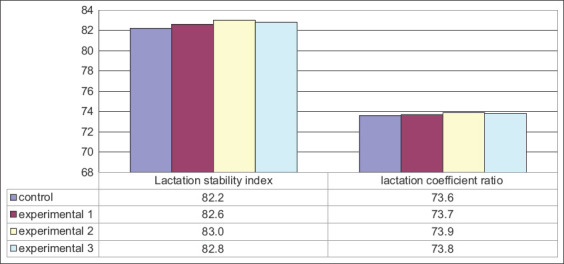
Lactation coefficients.

Since milk production during the entire lactation period was not even, lactation curves based on the average daily milk yields were constructed (Figure-2). Although the 1^st^ month values were approximately the same in all groups, the average daily milk yield of the control cows was reduced in the 2^nd^ month (34.15-34.20 kg). The daily milk yield in the control group was 36.80 kg by the 2^nd^ month, while the daily milk yields in the experimental groups were 0.97-2.16 kg (2.64-5.87%; p≤0.05-0.001) more. In the 3^rd^ and subsequent lactation months, there was a gradual decrease in the average daily milk yield of all groups. One of the main indicators of lactation evenness is the lactation stability index and the lactation efficiency indicator, which give a more detailed assessment of lactation curves (Figure-3).

All the animals involved in the experiment had stable milk yields throughout lactation. The milk yields were increased by 0.4% in Group 1, by 0.8% in Group 2, and by 0.6% in Group 3. The lactation efficiency ratio was increased by 0.1% in Group 1, 0.3% in Group 2, and 0.2% in Group 3.

Another essential production efficiency indicator is the quality of raw milk. The higher the milk quality, the higher the level of valuable ingredients, which reduces production costs, provides additional returns, and increases profitability. The energy supplement “Tanrem” affected milk quality (Table-3). The dry matter content in the control cows’ milk was lower than the experimental groups by 0.17-0.27% (p≤0.001) in summer and by 0.16-0.27% (p≤0.001) in winter. The level of non-fat milk solids was lower by 0.08-0.12% (p≤0.05) and 0.06-0.14% (p≤0.01), respectively.

**Table-3:** Chemical composition and quality of milk.

Indicator	Season of the year	Group			

		Control	Experiment 1	Experiment 2	Experiment3
Acidity, оТ	Summer	16.69±0.07	16.72±0.04	16.74±0.02*	16.74±0.04*
	Winter	16.90±0.05	17.01±0.06	17.06±0.05	17.06±0.06
Density, оА	Summer	26.97±0.18	27.44±0.14*	27.58±0.16*	27.54±0.14*
	Winter	28.69±0.06	28.86±0.01	28.96±0.16	28.90±0.17
Moisture, %	Summer	87.89±0.01	87.72±0.03***	87.62±0.06***	87.65±0.04***
	Winter	87.60±0.20	87.44±0.02***	87.33±0.02***	87.35±0.05***
Dry matter, %	Summer	12.11±0.01	12.28±0.03***	12.38±0.06***	12.35±0.04***
	Winter	12.40±0.02	12.56±0.02***	12.67±0.02***	12.65±0.05***
SOMO, %	Summer	8.44±0.03	8.52±0.01*	8.56±0.04*	8.55±0.03*
	Winter	8.63±0.03	8.69±0.03	8.77±0.02**	8.75±0.01**
Mass fraction of fat, %	Summer	3.67±0.02	3.76±0.02*	3.81±0.02***	3.80±0.02**
	Winter	3.77±0.03	3.86±0.04*	3.90±0.01***	3.90±0.04*
Mass fraction of protein, %	Summer	3.13±0.03	3.17±0.02	3.19±0.03	3.18±0.02
	Winter	3.25±0.03	3.28±0.3	3.32±0.01*	3.31±0.02
Lactose, %	Summer	4.63±0.01	4.65±0.01	4.66±0.01*	4.66±0.01*
	Winter	4.68±0.02	4.70±0.02	4.73±0.02*	4.72±0.01*
Ash, %	Summer	0.69±0.01	0.70±0.01	0.71±0.01*	0.71±0.01
	Winter	0.70±0.01	0.71±0.01	0.72±0.01	0.71±0.01
Calorie content, kcal	Summer	70.99±0.08	72.20±0.27***	72.86±0.41***	72.65±0.26***
	Winter	72.86±0.13	74.01±0.21***	74.71±0.10***	74.59±0.43**

The quality of milk obtained from the experimental cows in terms of fat and protein content exceeded similar indicators in samples collected from the control cows. Specifically, the protein content increased by 0.04-0.06% in summer milk and by 0.03-0.07% (p≤0.05) in winter milk. In addition, fat was increased by 0.09-0.14% (p≤0.05-0.001) and 0.09-0.13% (p≤0.05-0.001) due to the need for sufficient energy intake and activated metabolic processes in the animals’ bodies.

## Discussion

Manufacturers recommend the use of energy and carbohydrate additives in the diet of cows after calving and during early lactation. In this regard, the new high-carbohydrate energy supplement Tanrem was introduced into the diet of cows at a dose of 250-700 g/animal/day. Automated ration formulations were performed per Ventsova and Safonov, Torzhkov *et al*. [23,24]. The pasture diet was mainly comprised succulent fodder, while the indoo`r feeding consisted of roughages and concentrates. During the entire observation period, the proportion of succulent feed decreased with the introduced supplement. Animals receiving the high-carbohydrate energy additive were better able to digest nitrogen in their feed than the cows fed the control diet, with a statistical difference (p < 0.05) in the daily nitrogen balance of 6.6-19.6 g (2.7-8.2%). The cows consuming the studied supplement at a dose of 700 g/day/animal had the highest nitrogen utilization efficiency. Group 2 had moderately lower results. Mironova *et al*. [12] revealed that the energy supplement had the best effect on the nutrient and energy digestibility in the experimental cows.

The total protein level in the blood serum varied from 74.6-77.2 g/L in summer to 81.9-82.7 g/L in winter due to changes in feeding systems, nutrient composition in the feed, and reduced exercise in the winter. The biochemical composition of the blood serum was improved in the experimental cows, due to their higher dry mater absorption abilities, which also resulted in the higher total protein content of the experimental groups. The experimental animals had increased serum albumin levels and higher amounts of alpha- and gamma- globulins in winter, with beta-globulin content being the lowest. Changes in the quantitative composition of protein fractions make it possible to understand ongoing immune processes. The use of a new feed did not have a negative effect on animal immunity, which is consistent with the results of Andreeva *et al*. [10,13]. There was evident activation of biological protein synthesis and improved body defenses in response to changing weather conditions in the experimental groups. All data obtained were within physiological standards.

Milk yield is the most critical indicator of lactating cows. Lactation involves intense physiological and biochemical metabolic processes in the body due to the conversion of a large amount of energy and feed nutrients into milk. Additional energy sources are critical in the first 100 days of lactation in cows. Extra energy provides for milk synthesis without consuming the accumulated nutrient reserves in the body and the use of supplements does not decrease productivity. The second experimental group of cows (500 g supplement) had the highest milk productivity. The milk yield coefficient analysis revealed high milk productivity in all the studied groups, indicating the cows’ pronounced dairy type. A similar pattern was established by Safonov [11] and Mironova *et al*. [12].

Milk quality can also be evaluated by its density, which was higher in the experimental cows by 0.47-0.61°A (p≤0.05) in summer and by 0.17-0.27°A in winter. Similar seasonal dynamics was found in studies of new biologically active substances conducted by Gorlov *et al*. [25]. When comparing milk acidity values, there was a more pronounced seasonal variability, with increased acidity found in the winter: 0.21°T in the control sample, 0.29°T in Group 1, and 0.32°T in Groups 2 and 3. An intergroup analysis demonstrated higher acidity in the experimental samples by 0.03-0.05°T (p≤0.05) in summer and by 0.11-0.16°T in winter due to increased protein content. The milk acidity and density were within the regulatory limits set by the interstate standard for raw milk. Seasonal differences in milk composition and properties were previously studied by Kalaeva *et al*. [26] and Kakimov *et al*. [27]. The authors found a slight increase in milk acidity in the autumn-winter period, which can be explained by changes in animal nutrition. Higher nutrient content in the milk of the experimental animals increased its energy value. The caloric content of milk in the experimental samples increased by 1.21-1.87 kcal (1.70-2.63%; p≤0.001) in summer and by 1.15-1.85 kcal (1.57-2.54%; p≤0.01-0.001) in winter. Physicochemical milk characteristics in all the experimental groups varied by season and under the influence of the tested additive. The supplement had a positive effect due to the higher content of fat, protein, and dry matter, which increased the nutritional, biological, and energy values of the feed.

## Conclusion

The enrichment of dairy cattle diets with high-carbohydrate energy additives is very promising. Our tested feed additive improved nitrogen utilization and biochemical blood values, increased milk yields, and had a positive effect on the milk’s chemical composition and quality. Compared to the control cows, the experimental Holstein animals had higher total protein content, increased albumins, increased milk yields, higher dry matter concentrations, increased protein content, and higher fat contents. The most positive effect on these parameters was achieved using the Tanrem supplement at a dose of 500-700 g/­animal/day. It is important to note that high-quality feed preparation and proper diet formulations provide feed additives for specific physiological periods only. The studied energy supplement will not solve all the problems in dairy farming. Still, it will eliminate the negative energy balance after calving, maintain the health and welfare of the cows, increase milk yields, and bring in additional profit.

## Authors’ Contributions

AP: Initiated and developed the study under the guidance of IM. AN: Collected samples. EY: Examined milk chemical composition and quality. SI: Analyzed the data. IM: Drafted the manuscript. SI: Drafted and reviewed the manuscript. All the authors have read and approved the final manuscript.
